# A retrospective observational study comparing hair apposition technique, suturing and stapling for scalp lacerations

**DOI:** 10.1186/1749-7922-8-27

**Published:** 2013-07-25

**Authors:** Derya Ozturk, Bedriye Müge Sonmez, Ertugrul Altinbilek, Cemil Kavalci, Engin Deniz Arslan, Serhat Akay

**Affiliations:** 1 Numune Training and Research Hospital, Emergency Department, Ankara, Turkey

**Keywords:** Scalp laceration, Suturing, Stapling, Hair apposition, Emergency

## Abstract

**Aim:**

Scalp lacerations are commonly encountered in patients presenting to emergency department with trauma. Lacerations are repaired with suturing, stapling, adhesive tapes, and tissue adhesives. In this study, we aimed to compare the effectiveness of suturing, stapling, and hair apposition techniques used in repair of scalp lacerations in patients who presented to emergency department with scalp laceration.

**Materials and method:**

After obtaining approval of local ethics committee, we examined the effects of the three technique used to repair scalp lacerations on wound healing, complication rate, and patient satisfaction by recording data. Categorical variables were expressed as n and %. X^2^ test was used for statistical analysis. A p value less than 0.05 was accepted statistically significant.

**Results:**

Our study included a total of 134 patients of whom were treated 37 (27.6%) with hair apposition technique 49, 48 (35.8%) with suturing, and (36.6%) with stapling. There was a significant difference between the scalp repair technique and 7th and 15th day patient satisfaction rates in favor of the hair apposition technique (p < 0.05). There was a significant difference between the scalp repair technique and cosmetic problems after 15 days (p < 0.05). Cosmetic problems 15 days after the procedure were significantly lower in the hair apposition technique.

**Conclusion:**

In patients presenting to emergency departments with linear scalp laceration suturing, stapling, and hair apposition techniques can be safely applied. However, hair apposition technique has the advantages of being more satisfying, and having lower cosmetic problem and complication rates compared with other techniques.

## Introduction and objective

The main objective of wound repair is to restore skin integrity and, while doing this to reduce rate of infection, scarring, and functional impairment [1]. Lacerations are repaired with sutures, staples, adhesive tapes, and tissue adhesives. Each method has its own advantages and disadvantages [2]. Suturing is the most commonly used method in laceration repair [3]. It is the strongest of all wound closure materials and allows best approximation of wound edges irrespective of wound shape. However, it is also the most time-consuming and user-dependent among all techniques available. Repair via stapling is another method used for scalp lacerations. It is preferable to suturing in emergency services because it is a quicker and less painful procedure and associated with a lower cost and risk of needle stick injury to the operator. It is also preferred in pediatric age groups owing to the above-mentioned properties [4-6]. Hair apposition technique is an alternative technique in scalp lacerations. Hair apposition technique was first defined by Hock et al. in 2002. In this technique, 4–5 strands of hair are grasped on each side of the wound. These strands are crossed once and a drop of glue is placed where the strands cross to secure the wound [7]. In this study, we aimed to compare the effectiveness of suturing, stapling, and hair apposition techniques used in repair of scalp lacerations in patients who presented to emergency department with scalp laceration.

## Materials and method

This study was performed in a retrospectively at Numune Training and Research Hospital Emergency department between 01 January 2010 and 01 July 2010 after approval of the study by the local ethics committee (2010-33). Research carried out on humans must be in compliance with the Helsinki Declaration. Cosmetic problems, patient satisfaction, wound healing status, and complications were determined from the files of the patients who returned for follow-up examination on 7th and 15th days of suturing. After gathering data of the patients with scalp lacerations who were applied any of the hair apposition, suturing, or stapling methods. Method of wound closure was made to physician preference. Inclusion and exclusion criteria of the study given on Table 1. There was a used standart inclusion and exclusion criteria for three methods. Length and localization of the laceration, length of hair, the applied technique, satisfaction of the patient and complication parameters were recorded on study forms. Degree of pain in patients evaluated with visual analog scale (VAS). Infection was defined with redness and purulent wound drainage. Cosmetic problem defined according to doctor. The patients were divided into 3 groups as follows: Group 1, patients who were applied hair apposition technique; Group 2, patients who were applied suturing technique; and Group 3, patients who were applied stapling technique. Study data were analyzed using SPSS 15.00 software package. Categorical variables were expressed as n and %. X^2^ test was used for statistical analysis. A p value less than 0.05 was accepted statistically significant.

**Table 1:** Inclusion/ Exclusion criteria

**Inclusion criteria**	**Exclusion criteria**
Hair length of at least 1 cm	Nonlinear lacerations
Linear lacerations	Contaminated wounds
Laceration length shorter than 10 cm	Active arterial bleeding
Laceration repair carried out with the method of simple spaced percutaneous suturing using 4/0 monofilament polypropylene	Unstable vital signs or shock
Laceration repair carried out with stapling	Altered conscioussness
Laceration repair carried out with hair apposition and tissue adhesive	Irregular wound edges and associated tissue loss
	İmmunocompromised patient
	Comorbiditiy patient

## Results

Our study included a total of 134 patients of whom were treated 37 (27.6%) with hair apposition technique, 48 (35.8%) with suturing, and49 (36.6%) with stapling. The distribution of the technique according to patient demographics is given on Table 2.

**Table 2:** The distribution of the technique according to patient demografics

	**Hair apposition**	**Suturing**	**Stapling**	**p value**
Sex (Male/Female, n)	33/4	36/12	43/6	X^2^ = 4.04, p > 0.05
Age (mean ± SD)	31.68 ± 8.7	32.35 ± 9.5	32.02 ± 9.1	X^2^ = 0.10, p > 0.05

Distrubution of patient according to the technique and hair length was shown on the Table 3.

**Table 3:** Distrubution of the classified hair length and techniques used in the treatment of scalp laceration

**Hair lenght**	**Hair apposition (n)**	**Suturing (n)**	**Stapling (n)**	**p value**
Short (<3 cm)	12	20	25	X^2^ = 5.02, p > 0.05
Medium (3–6 cm)	17	14	15	
Long (>6 cm)	8	14	9	

**Figure 1:**
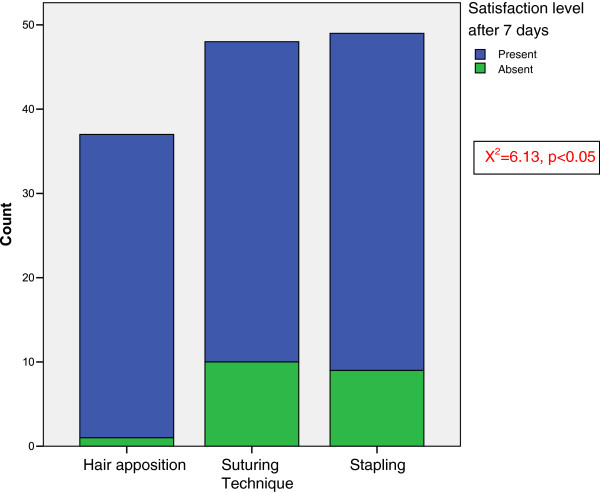
The graph of the relationship between the techniques and satisfaction level after 7 days.

A crosstabulation between the techniques used and the percentage of satisfaction after 7 days revealed that the latter was higher in hair apposition technique as compared with the other techniques (Figure 1). There was a significant relationship between the technique and satisfaction level after 7 days (X^2^ = 6.13, p < 0.05).

The most common complications after 7 days were redness and pain. These complications occurred most commonly in the suturing group (34.55% and 21.87%, respectively) followed by stapling technique (26.42% and 13.21%, respectively), and hair apposition technique (16.22% and 13.51%, respectively). The distribution of the complications 7 days after the procedure by the technique used is summarized on Table 4.

**Table 4:** Distribution of the complications on 7th day by the techniques used

	**Hair apposition**		**Suturing**		**Stapling**		**p value**
**Complications**	**n**	**%**	**n**	**%**	**n**	**%**	
**Pain**	5	13.51	12	21.87	7	13.21	X^2^ = 2.56, p > 0.05
**Serous wound drainage**	1	2.7	0	0	0	0	X^2^ = 2.61, p > 0.05
**Infection**	0	0.0	3	5.45	1	1.89	X^2^ = 3.05, p > 0.05
**Redness**	6	16.22	19	34.55	14	26.42	X^2^ = 5.54, p > 0.05
**Hair loss**	0	0	5	9.093	2	3.77	X^2^ = 4.78, p > 0.05
**Wound dehiscence**	1	2.7	0	0	3	5.66	X^2^ = 3.15, p > 0.05

**Figure 2:**
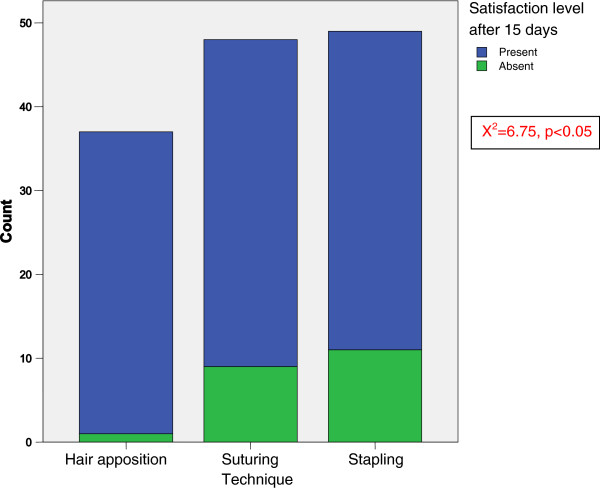
The graph of the relationship between the techniques and satisfaction level after 15 days.

There was a significant relationship between the technique and the satisfaction level after 15 days (X^2^ = 6.75, p < 0.05). According to this, satisfaction after 15 days depends on the technique used. The crosstabulation between the techniques used and satisfaction level after 15 days revealed that a stapling and suturing techniques were association with dissatisfaction whereas hair apposition technique was associated with much lower dissatisfaction rate (Figure 2).

**Figure 3:**
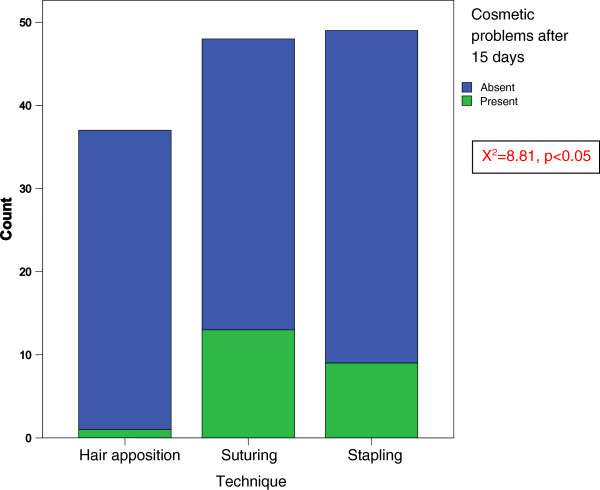
The graph of the relationship between the techniques and cosmetic problems.

The crosstabulation between the techniques used and the rate of cosmetic problems after 15 days revealed a higher rate of cosmetic problems in the suturing group than other groups (X^2^ = 8.81, p < 0.05) (Figure 3).

## Discussion

Emergency physicians can also employ hair apposition technique in addition to suturing and stapling in the treatment of scalp lacerations. In our study, hair apposition technique was associated with a higher rate of satisfaction than other techniques 7 days and 15 days after the procedure.

Hock et al., in a study where they used techniques of suturing and hair apposition in patients with scalp laceration, included lacerations up to 10 cm but did not mentioned about any relationship between the technique used and laceration length [7]. Both our study and previous studies suggested that a hair length of at least 1 cm is essential for application of hair apposition technique in scalp lacerations [7,8]. In our study there was no significant difference between the technique used and hair length.

Hock et al. compared complication and healing rates 7 days after treatment of scalp lacerations with suturing or hair apposition techniques and reported that wound healing and scar formation occurred more commonly in suturing whereas rates of infection or bleeding were not different in both groups [7]. Karaduman et al. used all three techniques in scalp lacerations and reported no cases of infection 7 days after the procedure. They reported abnormal hair growth in a patient who was treated with hair apposition, which was attributed by the authors to excess amount of glue used in the procedure, causing spread of glue in the wound [8]. Ong et al. used suturing or hair apposition in scalp lacerations and reported fewer complications 7 days after the procedure with the hair apposition technique [9]. Kanegaye et al., in a study on pediatric scalp lacerations, compared stapling and suturing with respect to complication rates 7 days after the procedure and reported fewer complications in stapling group [10]. We also found that the highest complication rate was with suturing. The most common complications 7 days after the procedure included redness, pain, and hair loss, which occurred most commonly with suturing followed by stapling and hair apposition techniques. The highest rate of infection was associated with suturing technique followed by stapling technique. Hair loss, an important cosmetic problem, occurred most commonly with suturing followed by stapling technique whereas hair apposition technique was not associated with hair loss 7 days after the procedure. Hock et al. reported a higher rate of satisfaction in patients treated with hair apposition technique compared with those treated with suturing technique. This high rate of satisfaction was related by the authors to the properties of the technique including quick application, less painful nature due to absence of need for anesthesia, and absence of need for shaving and suture removal [7]. Karaduman et al. applied all three techniques to their patients with scalp laceration and looked at patient satisfaction on day 30^th^. They reported a high rate of satisfaction in those who were applied hair apposition technique and 97% of patients would prefer this method in the event they sustained a scalp laceration in the future [8]. The rate of satisfaction was related with the technique used, such that patients were dissatisfied with stapling and suturing while dissatisfaction rate was quite low. In our study, Assessment of 7th and 15th day satisfaction rates revealed significant differences in favor of hair apposition technique. The painless nature of the technique and absence of suture removal may have increased patient satisfaction. In our study there was a significant association between the technique used and emergence of cosmetic problems 15 days later. We found that cosmetic problems were most prevalent in patients treated with suturing while they were least common in those managed with hair apposition technique. We think that this is because there was no need to shave hairs in this technique and we carefully placed only one drop of glue on the crossed strands without bringing the glue into contact with the wound. Otherwise excessive amount of glue will result in hair knots, leading to haircut while contact of tissue adhesive with laceration will result in decreased hair growth [11]. Kanegaye et al. compared the cosmetic outcomes of stapling or suturing in pediatric scalp lacerations 1 week after the procedure when they removed suture materials. They reported no cosmetic problems in stapling group [10].

In literature there are plenty of studies on application time of these techniques. Hock et al. compared suturing and hair apposition techniques with respect to application time and found that hair apposition technique was applied in a shorter time than other technique [7]. Kanegaye et al. reported that stapling technique was applied in a shorter time compared to suturing in pediatric patients with scalp laceration [10]. In a surgical study stapling and suturing techniques used in the treatment of long lacerations were compared in terms of application times. Stapling technique was reported to be associated with five-to-seven times shorter times compared with the suturing technique [12-15]. Karaduman et al., in a study examining the hair apposition and suturing techniques in emergency department patients with scalp laceration in terms of application times, reported that hair apposition technique was associated with shorter procedure time [8]. As our study was retrospective, we could not gather any information on application times. However, experience from our daily practice suggests that stapling method can be performed in a relatively shorter time.

Ong et al. compared hair apposition and suturing techniques in terms of treatment cost in scalp lacerations and reported that hair apposition technique had a significantly lower cost. They related that result to a shorter time of the procedure, absence of need for anesthesia and suture removal, and low complication rates. They expressed that the rate of scalp lacerations in EDs remain high and this technique would provide considerable cost saving [11]. Orlinsky et al., in a general study on costs of treatment of scalp lacerations in emergency departments, found that stapling was considerably advantageous with respect to overall cost [16]. We did not perform a cost analysis.

Hair apposition technique may be used more commonly in daily practice by virtue of its low complication and cosmetic problem rate coupled with high patient satisfaction rate. Determination of the ideal wound closure technique requires more prospective, randomized controlled studies with larger sample size that investigate factors effective on wound healing and satisfaction level.

## Limitations of the study

A major limitations of the study was a retrospectively of it. We could not gather any information on application times. As the social security institution of Turkey employs a per case payment system for suturing materials and procedure, no cost analysis was performed for any of the 3 groups.

## Conclusion

Emergency departments are one of the leading clinics where patient crowding is greatest. Thus, time-consuming procedures such as laceration repair may be problematic for the operators. In our study all these criteria were met by the hair apposition technique. Considering its low cost in addition to all these positive results, we feel that this technique will be used or preferred more frequently by physicians and patients in our country as the rest of the world.

## Competing interests

The authors declare that they have no competing interests.

## Authors’ contributions

DO: conception and design, or acquisition of data, or analysis and interpretation of data, have given final approval of the version to be published. BMS: acquisition of data, EA: revising it critically for important intellectual content; CK: analysis and interpretation of data or revising it critically for important intellectual content; EDA: have made substantial contributions to conception and design. SA: have made substantial contributions to conception and design. All authors read and approved the final manuscript.
